# Antimicrobial resistance profiles of *Escherichia coli* isolated from laying hens in Zambia: implications and significance on one health

**DOI:** 10.1093/jacamr/dlad060

**Published:** 2023-05-22

**Authors:** Steward Mudenda, Sydney Malama, Musso Munyeme, Scott Kaba Matafwali, Penjaninge Kapila, Patrick Katemangwe, Geoffrey Mainda, Andrew Nalishuwa Mukubesa, Mwendalubi Albert Hadunka, John Bwalya Muma

**Affiliations:** Department of Pharmacy, School of Health Sciences, University of Zambia, Lusaka, Zambia; Department of Disease Control, School of Veterinary Medicine, University of Zambia, Lusaka, Zambia; Department of Biological Sciences, School of Natural Sciences, University of Zambia, Lusaka, Zambia; Department of Disease Control, School of Veterinary Medicine, University of Zambia, Lusaka, Zambia; Clinical Research Department, Faculty of Infectious and Tropical Diseases, London School of Hygiene & Tropical Medicine, London, UK; Department of Disease Control, School of Veterinary Medicine, University of Zambia, Lusaka, Zambia; Department of Disease Control, School of Veterinary Medicine, University of Zambia, Lusaka, Zambia; Department of Veterinary Services, Central Veterinary Research Institute, Ministry of Fisheries and Livestock, Lusaka, Zambia; Department of Disease Control, School of Veterinary Medicine, University of Zambia, Lusaka, Zambia; Department of Animal Health, Centre for Infectious Disease Research in Zambia (CIDRZ), Lusaka, Zambia; Department of Disease Control, School of Veterinary Medicine, University of Zambia, Lusaka, Zambia

## Abstract

**Background:**

Antimicrobial resistance (AMR) has been deepening in the layer poultry sector in Zambia partly due to the inappropriate use of antimicrobials. *Escherichia coli* (*E. coli*), a commensal and zoonotic bacterium, can potentially be a source of AMR.

**Objectives:**

This study assessed the phenotypic AMR profiles of *E. coli* isolated from the apparent health-laying hens in Lusaka and Copperbelt provinces of Zambia.

**Methods:**

A cross-sectional study was conducted between September 2020 and April 2021 in which 365 cloacal swabs were collected from 77-layer farms based in Lusaka and Copperbelt provinces of Zambia. *E. coli* isolation and identification were done using cultural and biochemical properties and confirmed using the 16S rRNA gene sequencing. Antimicrobial susceptibility testing (AST) was done using the Kirby–Bauer disc-diffusion method. Data analysis was done using WHONET 2020 and Stata v.16.1.

**Results:**

Of the 365 samples, *E. coli* was isolated from 92.9% (*n* = 339). The AMR was detected in 96.5% (*n *= 327) of the isolates, of which 64.6% (*n *= 219) were multidrug-resistant (MDR). *E. coli* was highly resistant to tetracycline (54.6%) and ampicillin (54%) but showed low resistance to meropenem (0.9%), ceftazidime (6.2%) and chloramphenicol (8.8%).

**Conclusion:**

This study found a high prevalence of *E. coli* resistant to some commonly used antibiotics in poultry, which is a public health concern because of the potential contamination of eggs and layers of chicken meat that enter the food chain. Urgent attention is needed, including strengthening antimicrobial stewardship and surveillance programmes in layer poultry production in Zambia.

## Introduction

Antimicrobial resistance (AMR) has increased in poultry over the past decade due to the inappropriate use of antimicrobial agents.^1–4^ Antimicrobial agents have primarily been used for improved egg production, growth promotion, prophylaxis, metaphylaxis and therapeutics in the poultry industry.^5–8^ The use of antimicrobials in poultry production can be attributed to the increased demand for poultry products such as chicken meat and eggs.^9,10^ This has contributed to the continuous exposure of poultry microorganisms to antimicrobials and, thus, the development of AMR.^11,12^*Escherichia coli* (*E. coli*) are among the commensal or pathogenic microorganisms isolated from poultry that have become resistant to common antibiotics used in human and animal health.^13–16^*E. coli* causes infections such as urinary tract infections, bloodstream infections, sepsis and meningitis.^17–19^

In the poultry sector, the effect of AMR can cause economic losses due to challenges in containing antimicrobial-resistant infections, increased mortality, costs associated with the disposal of carcasses and the compromise on safety and security.^20^ In the public sector, AMR leads to increased medical costs, prolonged hospital stays and increased mortality.^21–23^ If this problem is not addressed, it is estimated to cause 10 million human deaths globally by the year 2050.^24–26^

Imprudent use of antimicrobials among poultry farmers worldwide have contributed to the emergence and spread of AMR.^27–30^ Studies have shown that most poultry farmers are unaware of the implications of inappropriate use of antimicrobials that leads to AMR.^31–33^ The ease of access to antibiotics without prescriptions has contributed to their inappropriate use.^31,34^ Poultry farmers’ high antibiotic demand often drives this to enhance production.^3,31,34^ This issue has been exacerbated by the ready availability and sale of antibiotics in drug stores and through street vendors.^34,35^ Additionally, antibiotics are incorrectly used in poultry to treat viral infections.^36^ Consequently, some poultry farmers administer lower doses of antibiotics to their chickens and usually stop before the course is completed, provided their birds look in fair health.^8^

At a global level, antimicrobial-resistant *E. coli* has been isolated from poultry.^37–40^*E. coli* was reportedly resistant to antimicrobials, including tetracycline, trimethoprim, ampicillin and gentamicin.^36,41,42^ Alongside the reported AMR profiles of *E. coli*, there are reports of multidrug-resistant (MDR) *E. coli* isolated from poultry.^41,43,44^ This is of public health concern and puts pressure on animal and human health prescribers when choosing antibiotics to prescribe for a particular condition. Additionally, antimicrobial-resistant *E. coli* can be transmitted from poultry to humans and cause infections that may be difficult or impossible to treat.^15,45–48^ As this problem can affect both humans and animals, there is a need to enhance the one health approach to address it.^49–52^

In Africa, some reports have been documented on the resistance patterns of *E. coli* isolated from poultry in some studies.^16,53–58^*E. coli* resistant to ampicillin, tetracycline, cotrimoxazole, ciprofloxacin and gentamicin has been identified. Besides, MDR *E. coli* isolated from poultry was reported from different studies, with 76% in Bangladesh,^15^ 78.1% in Ethiopia,^59^ 98.1% in Nigeria^60^ and 86.76% in Tanzania.^53^ This reported resistance to antimicrobials highlights the need for the prudent use in poultry farming.

In Zambia, antimicrobial-resistant *E. coli* have been isolated from humans,^61,62^ broiler chickens,^63,64^ dairy^65^ and wildlife and livestock.^66^ In broilers, *E. coli*, was observed to be resistant to tetracycline, ampicillin, cotrimoxazole and ciprofloxacin, whereas, in dairy, it was resistant to tetracycline, ampicillin, cotrimoxazole and ciprofloxacin.^65^ Besides, *E. coli* isolates from wildlife and livestock were highly resistant to ampicillin (27%), ceftazidime (14.3%), cefotaxime (9.5%) and kanamycin (9.5%).^66^ In these animal species, a prevalence of 36.5% MDR was reported in broilers^63%^ and 18.8% in wildlife.^66^ However, the study of the prevalence and AMR profiles of *E. coli* isolated from laying hens in Zambia was not reported prior to this study.

The reported AMR in Zambia requires enhancement and implementation strategies to address this problem. Among the proposed strategies, the Zambia Multi-sectoral National Action Plan (NAP) on AMR, launched in 2017, aimed at addressing this problem in humans and animals.^67,68^ The NAP on AMR was developed in line with the Global Action Plan on AMR to successfully prevent and treat infections across all populations with safe and effective medicines and reduce AMR.^69^ The NAP on AMR strives to address AMR in animals and humans by increasing awareness and knowledge of AMR among different populations and promoting the rational use of antimicrobials.^67^ Alongside this, strengthening antimicrobial stewardship (AMS) and surveillance programmes are critical in addressing AMR across humans and animals.^67,69–74^ This study assessed the AMR profiles of *E. coli* isolated from laying hens in Lusaka and Copperbelt provinces of Zambia.

## Materials and methods

### Study design, site and population

**Figure 1.**
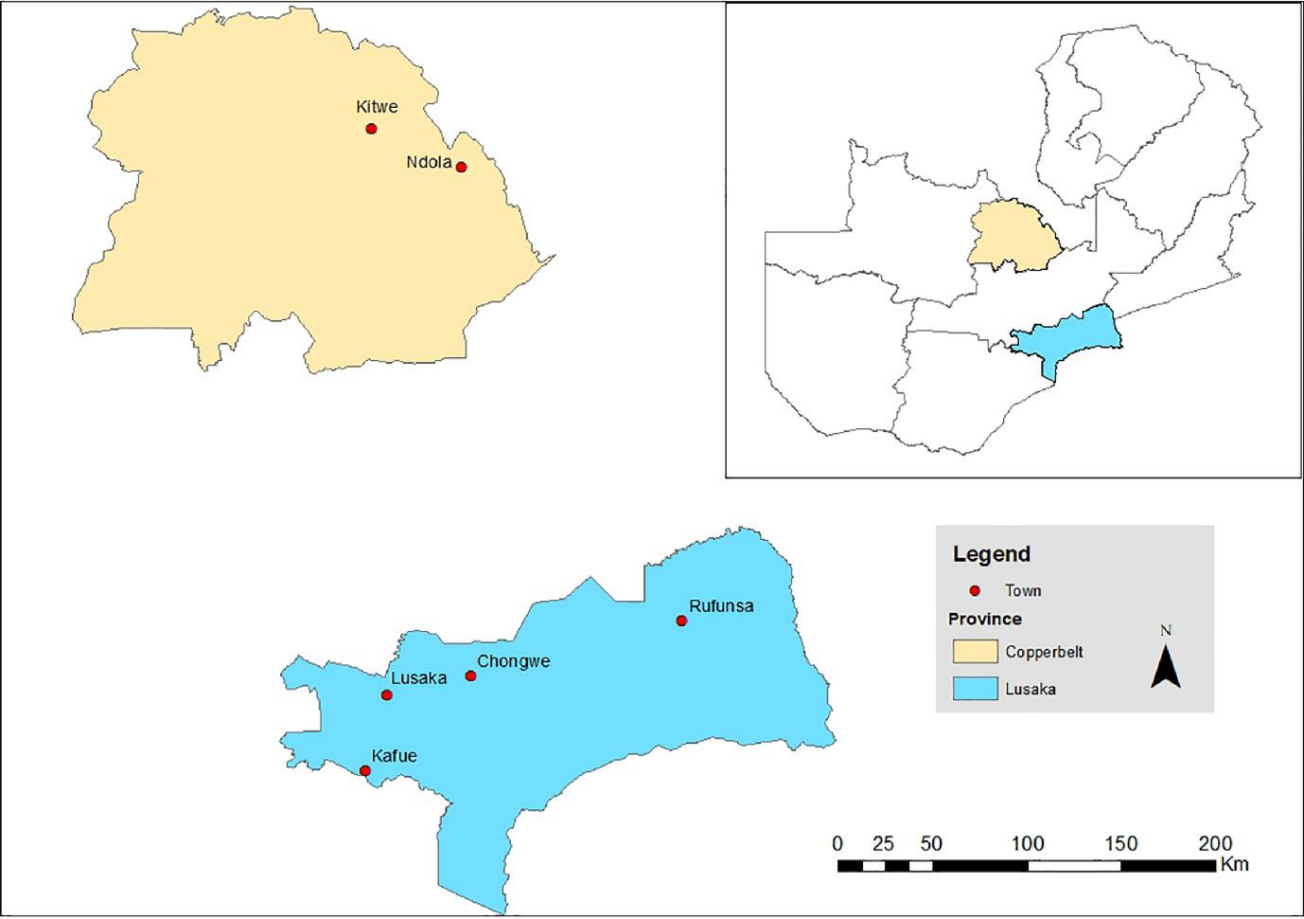
Map of Zambia indicating the study sites in Lusaka and Copperbelt provinces (ARCGis: 2021).

A cross-sectional study was conducted in layer farms of Lusaka and Copperbelt provinces from September 2020 to April 2021. The two provinces contribute (75%) to poultry production in the country.^75^ On the basis of information from the Poultry Association of Zambia, Lusaka province contributes 50% while Copperbelt province contributes 25% to poultry production.^75^ The study sites are shown in Figure 1.

### Study population and sampling

This study enrolled layer poultry farmers after obtaining informed consent. The study participants were poultry farmers who were rearing laying hens and resided in Zambia's Lusaka and Copperbelt provinces. A multi-stage sampling method was used to select six districts, farms and later laying hens from the poultry houses. All layers in the production age were sampled randomly to increase the chances of all hens being selected. However, laying hens that were on antibiotic treatment or sick during data collection were excluded from the study. We used registers from the District Veterinary Offices and the Poultry Association of Zambia to identify the layer poultry farmers. Farm visits were conducted with the help of Veterinary Assistants. The layer poultry farms were categorized into small-scale (farmers rearing up to 1000 hens), medium-scale (farmers rearing 1001 to 10 000 hens) and large-scale (farmers rearing greater than 10 000 hens). On the basis of the registers from the District Veterinary Offices and the Poultry Association of Zambia, active layer poultry farmers were estimated to be 96, i.e. 56 from Lusaka and 40 from the Copperbelt province. A recent study also reported this estimated population of layer poultry farms.^31^ Before sampling, the sample size was estimated using Ausvet Epitools (https://epitools.ausvet.com.au/) at a 95% confidence level, 50% estimated proportion and a 5% desired precision, as reported in a similar poultry study.^76^ Because there were few layer poultry farms that were identified and active in rearing laying hens, we conducted a complete enumeration to recruit all the farmers, which resulted in the recruitment of 77-layer poultry farms.

At each farm, laying hens were randomly sampled per 25 m^2^ (sampling unit) from each poultry house. A cloacal swab was collected from each laying hen per sampling unit and pre-enriched in 10 millilitres (mL) of buffered peptone water (BPW) broth (Oxoid, Basingstoke, UK). The pre-enriched samples were then transported within eight hours of collection to the Public Health Laboratory at the School of Veterinary Medicine at University of Zambia, for processing and analysis. A total of 365 cloacal swab samples were collected and processed for *E. coli* isolation.

### Isolation and identification of E. coli isolates

To isolate *E. coli*, the samples were incubated in the pre-enriched BPW broth at 37˚C for 16–24 hours. Aliquots of the pre-enriched broth were then spread and cultured on MacConkey agar (Oxoid, Basingstoke, UK) plates and incubated aerobically at 37˚C for 24 hours. The pink-coloured (lactose fermenting) colonies were sub-cultured on Eosin Methylene Blue (EMB) agar (Oxoid, Basingstoke, UK) plates and incubated aerobically for an additional 24 hours. The green metallic colonies on EMB were presumed *E. coli* and were sub-cultured on nutrient agar (Oxoid, Basingstoke, UK) plates and incubated aerobically for 24 hours. The *E. coli* colonies appeared large, thin, circular and greyish-white on nutrient agar. Further identification of the isolates was made using the Analytical Profile Index (API 20E) (Biomerieux^®^, Inc., 100 Rodolphe Street, Durham, NC 27712, USA) test kit. We stored the presumptive *E. coli* isolates in 10% glycerol at −20˚C before further analysis.

### Confirmation of E. coli isolates

The presumptive *E. coli* isolates were sequenced using 16S rRNA as described for *E. coli*.^77^ The boiling method was used to extract DNA from presumptive *E. coli* isolates, as was used in a similar study.^78^ Each pure colony was suspended in 200 µL of nuclease-free water and heated at 95°C for 5 minutes. The suspension was centrifuged at 6000 *g* for 2 minutes at four °C to extract the DNA. Polymerase Chain Reaction (PCR) was used for DNA amplification using *Taq* polymerase, and the *uidA* F (Forward) primers (5′-CGGAAGCAACGCGTAAACTC-3′) and *uidA* R (Reverse) primers (5′-TGAGCGTCGCAGAACATTACA-3′) (Sigma-Aldrich, Merck, Germany) in a thermo-cycler. The PCR conditions were; initial denaturation 95°C for 3 minutes, and 40 cycles of 95°C for 45 seconds, 55°C for 45 seconds and 72°C for 60 seconds. The final extension was at 72°C for 5 minutes and held at 4°C. After that, the amplicons were run on 1.5% agarose gels by gel electrophoresis in 1X tris-acetate EDTA (TAE) buffer at 100 volts for 30 minutes. The gels were then stained with ethidium bromide and read on a BioDoc-IT™ Imaging System Trans-illuminator to confirm the amplifications of the *uidA* target region.

### Antimicrobial susceptibility testing

Antimicrobial susceptibility testing (AST) was done using a panel of 13 antibiotics by the Kirby disc-diffusion method. The AST procedure was done as described in other studies.^79–81^ The antibiotic discs (Oxoid, Basingstoke, Hampshire, UK) that were used included amoxicillin/clavulanic acid (20/10 µg), ampicillin (10 µg), cefepime (30 µg), cefotaxime (30 µg), ceftazidime (30 µg), chloramphenicol (30 µg), ciprofloxacin (5 µg), gentamicin (10 µg), meropenem (10 µg), nalidixic acid (30 µg), nitrofurantoin (300 µg), tetracycline (30 µg) and trimethoprim/sulfamethoxazole (1.25/23.75 µg).

The bacterial suspension was prepared and turbidity adjusted to 0.5 McFarland standard, after which it was inoculated onto Mueller-Hinton Agar (Oxoid, Basingstoke, Hampshire, UK) petri dishes and incubated at 37°C for 16–24 hours. The zones of inhibition were measured in millimetres using a Vernier calliper, and results were interpreted according to the Clinical and Laboratory Standards Institute (CLSI) 2020 guidelines as susceptible (S), intermediate (I) and resistant.^82^ We used *E. coli* ATCC 25922 for quality control.

### Data process and analysis

Data analysis used Stata version 16.1/BE (Stata Corp., College Station, TX, USA) and WHONET 2020. The zones of inhibition were interpreted using the CLSI 2020 guidelines and interpreted as susceptible (S), intermediate (I) and resistant (R). Frequencies and percentages are presented in Tables 1 and 2.

### Ethical approval

We obtained ethical clearance from the ERES Converge Ethics Committee with a protocol ID: reference no. 2019-Dec-004. Permission to conduct the research in the selected sites was also obtained from the Zambia National Health Research Authority. Additionally, we obtained further permission to collect data in layer poultry farms from the Lusaka and Copperbelt Provincial and District Veterinary Offices. Finally, we obtained informed consent from the layer poultry farmers to collect samples from their laying hens.

## Results

Seventy-seven layer poultry farms from six districts across Lusaka and Copperbelt provinces were enrolled in this study from which 365 cloacal swabs were collected from the laying hens. Of the 365 cloacal swab samples, 339 tested positive for *E. coli*, resulting in a 92.9% positivity rate (Table 1).

**Table 1.** Distribution of samples collected from laying hens

Province	District	Number of farms sampled, n (%, 95% CI)	Number of samples collected	Samples that yield *E. coli* isolates	Positivity rates (%)
Lusaka	Chongwe	17 (22.1; 14.0–33.0)	103	96	93.2
	Kafue	20 (26.0; 17.2–37.1)	81	68	84.0
	Lusaka	5 (6.49;2.67–14.9)	34	34	100
	Rufunsa	3 (3.90; 1.23–11.7)	7	7	100
Copperbelt	Kitwe	22 (28.6; 19.4–39.9)	94	88	93.6
	Ndola	10 (13.0; 7.03–22.7)	46	46	100
	**Total**	**77**	**365**	**339**	

*E. coli* isolates were highly resistant to tetracycline (54.6%) and ampicillin (54.0%) while highly susceptible to meropenem (94.7%), chloramphenicol (85.8%) and ceftazidime (85.3%) as depicted in Table 2.

**Table 2.** AMR patterns of *Escherichia coli* isolates (*n* = 365)

Antibiotic name	% R	% I	% S	% R 95%CI
Amoxicillin/Clavulanic acid	25 (7.4)	32 (9.4)	282 (83.2)	13.1–21.3
Ampicillin	183 (54.0)	40 (11.8)	116 (34.2)	48.5–59.4
Cefotaxime	103 (30.4)	39 (11.5)	197 (58.1)	25.6–35.6
Ceftazidime	21 (6.2)	29 (8.6)	289 (85.3)	4.0–9.5
Cefepime	21 (6.2)	61 (18.0)	257 (75.8)	4.0–9.5
Chloramphenicol	30 (8.8)	18 (5.3)	291 (85.8)	6.1–12.5
Ciprofloxacin	86 (25.4)	80 (23.6)	173 (51.0)	20.9–30.4
Gentamicin	29 (8.6)	69 (20.4)	241 (71.1)	5.9–12.2
Meropenem	3 (0.9)	15 (4.4)	321 (94.7)	0.2–2.8
Nitrofurantoin	41 (12.1)	72 (21.2)	226 (66.7)	8.9–16.2
Tetracycline	184 (54.3)	52 (15.3)	103 (30.4)	49.1–59.9
Trimethoprim/Sulfamethoxazole	90 (26.5)	12 (3.5)	237 (69.9)	22.0–31.6
Nalidixic acid	82 (24.2)	58 (17.1)	199 (58.7)	19.8–29.2

Note. R = Resistant, I = Intermediate, S = Susceptible, 95% CI = 95% confidence interval.

### Multidrug-resistant, extensively drug-resistant and pan-drug-resistant E. coli

Overall, 12/339(3.54%; 95% CI: 1.93–6.27) of the isolates were susceptible to all antibiotics, while 327/339 (96.5; 95% CI: 93.73%–98.07%) were resistant to at least one antibiotic. A total of 219/339 (64.6%; 95% CI: 59.22–69.64) isolates were MDR and resistant to three or more antibiotics from different classes. Of these isolates, 25/339 (7.37%; 95% CI: 4.92–10.83) were possible XDR isolates. However, no PDR isolates were recorded.

Overall, layer poultry farms obtained MDR isolates from 75/77(97.4%; 95% CI: 89.9–99.4). All the farms (45/45) from Lusaka province and 30/32 (93.8%; 95% CI: 77.4–98.5) from the Copperbelt province recorded MDR isolates.

The most MDR pattern was seen with resistance to AMP, CTX and CIP, while the least frequent pattern was seen with resistance to AMP, CTX, FEP and CHL (Table 3).

**Table 3.** Selected common and less common MDR patterns of Escherichia coli

Antimicrobial combination	Number of isolates	Number of antimicrobial classes
AMC, AMP, CTX, CIP	4	3
AMC, AMP, CTX, FEP, CIP	4	3
AMC, AMP, CTX, CAZ, CIP	3	3
AMC, AMP, CTX, FEP, CHL, CIP	4	4
AMP, CTX, CAZ, FEP, CIP	6	3
AMP, CTX, FEP, CHL	2	3
AMP, CTX, FEP, CIP	12	3
AMP, CTX, CHL, CIP	4	4
AMP, CTX, CHL	3	3
AMP, CTX, CIP	19	3
AMP, CAZ, FEP, CIP	3	3
AMP, CAZ, CIP	5	3
AMP, CHL, CIP	5	3
AMC, AMP, CTX, CAZ, FEP, CHL, CIP	5	4
AMC, AMP, CTX, CAZ, FEP, CIP	6	3

Note: AMC = Amoxicillin/clavulanic acid; AMP = Ampicillin; CTX = Ceftriaxone; FEP = Cefepime; CHL = Chloramphenicol; CIP = Ciprofloxacin; CAZ = Cefpodoxime.

## Discussion

In this study, we assessed the AMR profiles of *E. coli* isolated from laying hens in selected districts of Lusaka and Copperbelt provinces in Zambia. Our study found a high isolation rate of 92.9%, with a 96.5% prevalence of *E. coli* resistant to at least one antibiotic. Additionally, 64.6% of the isolates were MDR. This study found that most *E. coli* isolates were highly resistant to tetracycline (54.6%), ampicillin (54.0%), cefotaxime (30.4%), trimethoprim-sulfamethoxazole (26.5%), ciprofloxacin (25.4%) and nalidixic acid (24.2%). Conversely, *E. coli* was highly susceptible to meropenem (94.7%), chloramphenicol (85.8%), ceftazidime (85.3%), amoxicillin-clavulanic acid (83.2%) and cefepime (75.8%).

The present study found a high *E. coli* isolation rate of 92.9%. The isolation rate of *E. coli* in our study was similar to (93%) what was reported in Sierra Leone.^83^ However, our isolation rate was slightly higher than (86%) what was reported in broiler chickens in Bangladesh.^15^ Other studies have reported even higher isolation rates, including 100% in Tanzania,^53^ 99% in the USA^84^ and 94% in Nepal.^85^ The high isolation rate of *E. coli* reported in our study and similar studies could be due to adherence to the microbiology isolation protocols that resulted in increased bacteria recovery. This level of isolation rate makes *E. coli* a suitable microorganism to evaluate the AMR prevalence and profiles in many production systems, including layer poultry farms. However, lower isolation rates have been reported in other studies, such as a study in Bangladesh that used frozen chicken samples.^41^ The differences in sample sources may account for these variations in isolation rates.

The highest resistance of *E. coli* to antibiotics was observed with tetracyclines. A previous study conducted from commercial farms in the Chisamba district of Zambia reported a 100% resistance of *E. coli* to tetracycline.^86^ Another recent study in Zambia found high (87.9%) resistance of *E. coli* to tetracycline.^87^ The ease of access to tetracyclines in Zambia's poultry industry without prescriptions may contribute to AMR.^88^ Our findings corroborate observations from other studies conducted on poultry.^13,41,55,59,89^ The misuse of tetracyclines in poultry for growth promotion, improved egg production, prophylaxis and treatment of diseases has contributed to the resistance of *E. coli* to these drugs.^90–93^ However, by contrast, a study in Bangladesh found that *E. coli* isolated in poultry were highly resistant to levofloxacin.^15^ These discrepancies may be due to differences in poultry disease burdens and the availability of particular poultry antimicrobials across countries. Furthermore, the ease of access to common antibiotics such as tetracyclines, quinolones and penicillins for use in poultry has contributed to development and spread of AMR.^42,53^

Our study found that *E. coli* was also highly resistant to ampicillin. Our findings are similar to a study that was conducted in poultry farms in Zambia.^87^ Additionally, our findings are consistent with those reported in other studies.^14,53,60,85,94^ The resistance of *E. coli* to ampicillin may indicate the misuse of penicillins in the poultry sector. Additionally, studies in humans have also reported high resistance of *E. coli* to ampicillin.^61,62,95,96^ The resistance rate reported in our study is higher than that reported in a study that was done in the USA^97^ and in Sierra Leone.^83^

This study found that *E. coli* was resistant to cefotaxime, a third-generation cephalosporin. A similar resistance rate to cefotaxime has been reported in Bangladesh,^15^ and resistance to ceftiofur, another third-generation cephalosporin, was reported in China.^98^ Studies suggest that increased resistance of *E. coli* to third-generation cephalosporins may be associated with the administration of beta-lactams, which may result in the production of extended spectrum beta-lactamases.^98–100^ In human medicine, third-generation cephalosporins such as cefotaxime and ceftriaxone are ‘Watch group’ antibiotics and should only be used when the ‘Access (to first-line) group’ have failed.^101^ However, there is evidence that antibiotics such as ceftriaxone or cefotaxime are usually prescribed inappropriately, thereby increasing the risk of bacterial resistance to cephalosporins.^102–104^

In Zambia, the misuse of enrofloxacin, a quinolone commonly used in poultry, is evidenced by the resistance rates of *E. coli* to ciprofloxacin and nalidixic acid in our present study, a recent comparable study^87^ and the ease of access to poultry antibiotics,^33,88^ in Zambia. This finding aligns with similar resistance patterns of *E. coli* to quinolones reported in other studies.^15,16,44,85,105^ The misuse of enrofloxacin in the poultry sector may contribute to the resistance of *E. coli* and other microorganisms to this drug, as well as other quinolones.^106^ This highlights the importance of prudent use of antimicrobials in the poultry sector to prevent the development and spread of AMR that would subsequently compromise the treatment of human infections.

Our study found high resistance (96.5%) of *E. coli* to at least one of the tested antibiotics, similar to a study done in China, where 94% of *E. coli* isolates were resistant to at least one antibiotic.^44^ This high resistance may be partially attributed to the inappropriate use of antibiotics in poultry.^33^ The detection of 64.6% MDR *E. coli* in our study is a public health concern requiring urgent attention and control of antibiotic use in poultry. Similar findings were reported in other studies. An earlier study from three commercial poultry farms in Zambia showed that 4.8% of *E. coli* were MDR,^86^ while higher levels of MDR *E. coli* have been reported in broiler chickens in Bangladesh (100%),^41^ 95.7% in Austria,^107^ 86.76% in Tanzania,^53^ 76% in Bangladesh,^15^ 71% in Nepal,^85^ 56.3% Nigeria^108^ and 44% in Ireland.^37^ The treatment failure of poultry diseases due to MDR microorganisms can cause farmers to seek more options of the available antimicrobials that can further increase the selection pressure of AMR and make the treatment of infections even more difficult or impossible.^109–111^ The occurrence of MDR isolates in our study, as well as in other studies, highlights the need to intensify biosecurity measures in poultry^10,108,112–117^ and for more robust AMS and surveillance programmes to address AMR.^118–125^

This study highlights the AMR profiles of *E. coli* isolated from laying hens from Zambia’s two provinces that produce the largest number of poultry products. However, this study focused on one priority microorganism and does not give a clear picture of the resistance patterns of other priority microorganisms isolated from poultry.

### Conclusion

This study found a high resistance of *E. coli* to antibiotics commonly used in both poultry and humans in Zambia. The presence of MDR isolates is a significant public health concern because of the potential risk of transmission of AMR from chickens and eggs to humans. The regulation of antibiotic use in poultry is critical in addressing this issue. To combat this problem, there is a need to increase education and awareness among poultry farmers and veterinary drug dispensers on the rational use of antimicrobials, biosecurity measures, vaccinations and AMR. Furthermore, a multifaceted response, implementation and strengthening of AMS and surveillance programmes in poultry should be promoted to reduce the development and spread of AMR.
